# The efficacy of the traditional Korean herbal medicine *Tongsayobang* for the treatment of irritable bowel syndrome: A protocol for a systematic review and meta-analysis

**DOI:** 10.1097/MD.0000000000028116

**Published:** 2021-12-03

**Authors:** Gajin Han, Seok-Jae Ko, Keumji Kim, Hyejin Jun, Jae-Woo Park

**Affiliations:** a Kyung Hee Sweet & Sunny Korean Medicine Clinic, Gyeonggi-do, Republic of Korea; b JINRESEARCH, Gyeonggi-do, Republic of Korea; c Department of Gastroenterology, College of Korean Medicine, Kyung Hee University, Seoul, Republic of Korea; d Department of Internal Medicine, Kyung Hee University Hospital at Gangdong, Seoul, Republic of Korea.

**Keywords:** Irritable bowel syndrome, randomized controlled trial, systematic review, *Tongsayo-bang*

## Abstract

**Background:**

Irritable bowel syndrome (IBS) is a functional bowel disorder with symptoms of recurrent abdominal pain associated with a change in stool frequency or appearance that decreases patient quality of life. Conventional Western medicine has limited efficacy in treating IBS. *Tongsayobang* (TSYB) is a traditional Korean medicine that has been used to treat lower intestinal problems. This study provides a procedure for conducting a systematic review of the efficacy and safety of TSYB for IBS.

**Methods and analysis:**

The main electronic databases will be searched up to May 2021 for randomized controlled trials and quasi-randomized controlled trials evaluating the effect of TSYB or modified TSYB on patients with IBS. The primary outcome will be the overall efficacy rate. The secondary outcome will be data such as IBS-related quality of life, global symptom scores, and adverse events. This study will adopt the Preferred Reporting Items for Systematic Reviews and Meta-Analyses statement, and will involve a meta-analysis, if possible. The methodological quality of the included studies will be assessed using the Risk of Bias tool from the Cochrane Handbook, version 6.1.0.

**Ethics and dissemination:**

Ethical approval is not required because this study does not include any participants’ personal information.

**OSF registration number:**

DOI 10.17605/OSF.IO/M32BK (https://osf.io/m32bk)

## 1 Introduction

The Rome IV criteria define irritable bowel syndrome (IBS) as a functional bowel disorder with symptoms of recurrent abdominal pain occurring at least once per week, on average, in the previous 3 months, with a duration of at least 6 months. Abdominal pain is related to defecation or is associated with changes in stool frequency or appearance.^[1]^

IBS is one of the most common functional gastrointestinal disorders, affecting 3% to 5% of the world population,^[2]^ and it imposes significant socioeconomic burdens as it affects patients’ quality of life.^[3]^

The pathogenesis of IBS is complex and various, including abnormal gastrointestinal motility, visceral hypersensitivity, changes in the gut-brain axis, psychosocial stressors, and immune dysregulations.^[4]^ The treatment of IBS includes diet, antispasmodics, antidiarrheal agents, antidepressants, laxatives, biofeedback, probiotics, and psychological treatments. Although these treatments relieve symptoms in some patients with IBS, they have limited efficacy, and the recurrence rate of IBS is high. Thus, other effective treatment methods for IBS^[5]^ are required. In this context, many patients in Korea tend to seek complementary and alternative medicine. Moreover, patients who are satisfied with conventional therapy have also been interested in complementary and alternative medicine treatments, such as herbal medicines, acupuncture, dietary supplements, and mind-body-based interventions.^[6]^

*Tongsayobang* (TSYB) has traditionally been used for the treatment of IBS. TSYB, which was officially documented in the *Jingyue Complete Works* in 1624, is composed of four herbs:^[7]^ Paeoniae Radix Alba, Saposhnikovia Divaricate, Pericarpium Citri Reticulatae, and Atractylodes Macrocephala. TSYB is used for the treatment of chronic diarrhea and abdominal pain.^[8]^ In vivo studies showed that TSYB can relieve smooth muscle contraction and improve visceral hypersensitivity by regulating 5-hydroxytryptamine and substance P in colonic tissues.^[9–11]^

However, there has been a lack of systematic verification of the efficacy and safety of *Tongsayobang* for IBS to support the clinical evidence. This systematic literature review will analyze and evaluate the efficacy and safety of TSYB for IBS and provide evidence for the applicability of TSYB as a complementary treatment for IBS.

## 2 Methods

### 2.1 Study registration

A protocol has been registered on the OSF registries (URL: https://osf.io/m32bk). This systematic review will be performed according to the Preferred Reporting Items for Systematic Reviews and Meta-Analysis.

### 2.2 Inclusion criteria for study selection

#### 2.2.1 Types of studies

The systematic review will include randomized controlled trials (RCTs) and quasi-RCTs. Study designs other than RCTs, such as retrospective studies, case reports, pre-clinical/animal studies, literature research, qualitative research, review studies, and conference presentations, will be excluded.

#### 2.2.2 Types of patients

The subjects of our study will be IBS patients treated with TSYB, with no restrictions on ethnicity, nationality, sex, age, or biological status. The ROME diagnostic criteria (Rome I–IV) will be used for IBS diagnosis. Studies in which IBS patients were diagnosed with other organic intestinal diseases, such as Crohn disease, colorectal cancer, or ulcerative colitis, will be excluded.

#### 2.2.3 Types of interventions

Studies using TSYB and modified TSYB for the treatment of IBS will be included. TSYB with any formulation administered orally, such as decoction, capsules, tablets, pills, and powders, will be considered as experimental interventions. There will be no limitations on the dosage of medicine or the frequency, or duration of treatment. The comparator groups will include patients taking a placebo of TSYB and conventional Western medicines, such as antispasmodic agents.

#### 2.2.4 Types of outcome measures

The primary outcome will be the overall efficacy rate. The secondary outcome will be IBS-related quality of life, global symptom scores, adverse events, and other such data.

### 2.3 Data source and data collection procedures

#### 2.3.1 Databases

We will search 11 electronic databases for studies, without language restrictions, from their inception until September 2021. The databases include 4 English databases (Medline [via PubMed], EMBASE, the Cochrane Central Register of Controlled Trials [CENTRAL], and the Allied and Complementary Medicine Database [AMED]), 5 Korean databases (the Korean Studies Information Service System, National Digital Science Library, Korean Medical Database, KoreaMed, and Oriental Medicine Advanced Searching Integrated System), 1 Chinese database (China National Knowledge Infrastructure), and 1 Japanese database (Citation Information by NII). The combination of “irritable bowel syndrome” and “*Tong Sa Yo Bang*” will be used as the search term, with modifications for individual searches to align with the instructions of different databases. The search strategy for Medline via PubMed is presented in Table 1.

**Table 1:** Strategy for searching Medline via PubMed.

#1	Irritable Bowel Syndrome[mh] OR “Irritable Bowel Syndrome” OR “irritable Bowel Syndromes” OR “Syndrome, Irritable Bowel” OR “Syndromes, Irritable Bowel”
#2	“Colon, Irritable” OR “Irritable Colon”
#3	“Colitis, Mucous” OR “Colitides, Mucous” OR “Mucous Colitides” OR “Mucous Colitis”
#4	“Colonic disease, functional” OR “Irritable Bowel” OR “Spastic colon” OR “functional bowel disease” OR “functional colonic disease” OR Colonic Diseases, Functional[mh]
#5	“irritable bowel syndrome”[tw] OR irritable bowel syndrome∗[tw] OR IBS[tw] OR “functional abdominal dpain”[tw] OR “functional gastrointestinal disorders”’[tw]
#6	#1 OR #2 OR #3 OR #4 OR #5
#7	“Tong Sa Yo bang” OR Tong-Xie-Yao-Fang OR “Tongxie Yaofang” OR “Tong Xie Yao Fang” OR Modified-Tong-Xie-Yao-Fang OR “Modified Tongxie Yaofang” OR “Modified Tong Xie Yao Fang” OR Jiawei-Tong-Xie-Yao-Fang OR “Jiawei TongXie YaoFang” OR “Jiawei Tongxieyaofang”
#8	#6 AND #7

#### 2.3.2 Study selection and exclusion

**Figure 1:**
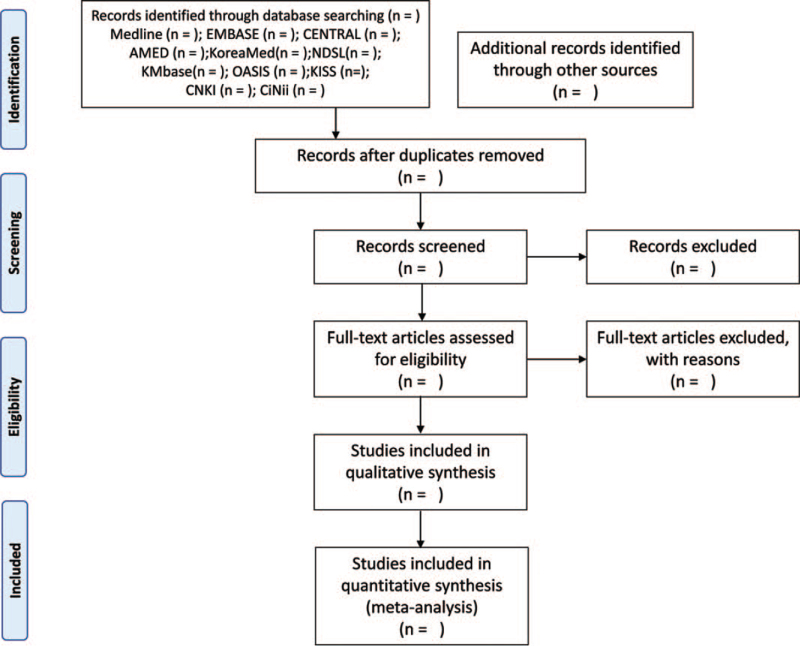
PRISMA flow diagram of the literature screening and selection process. AMED = Allied and Complementary Medicine Database, CENTRAL = Cochrane. Central Register of Controlled Trials, CiNii = Citation Information by Nii, CNKI = China National Knowledge Infrastructure, KISS = Korean Studies Information Service System, KMbase = Korean Medical Database, NDSL = National Digital Science Library, OASIS = Oriental Medicine Advanced Searching Integrated System, PRISMA = Preferred Reporting Items for Systematic Reviews and Meta-Analyses.

The titles, abstracts, and full texts of the searched studies will be screened by two independent researchers (GH and SJK) to identify the articles that meet the inclusion criteria. The final selected studies will be confirmed through an agreement between the 2 researchers. If there is a disagreement, an independent third party (JWP) will be invited to discuss the issue. The reasons for exclusion and number for excluded studies will be presented in the Preferred Reporting Items for Systematic Reviews and Meta-Analysis diagram (Fig. 1).

#### 2.3.3 Data extraction

Two independent reviewers will screen and assess the eligibility of all retrieved studies and extract data from the included studies using a predefined data acquisition form. This form will include four main domains: general information (title, authors, country of study, journal, year of publication), participant characteristics (age, sex, diagnostic criteria), details of the intervention and comparisons (number of participants in each group, formulation and type of TSYB, medicine dosage, comparisons, treatment frequency or duration, follow-up information), and outcomes (primary and secondary outcomes, adverse events). The data on TSYB will be extracted according to the 2017 Consolidated Standards of Reporting Trials Extension for Chinese Herbal Medicine Formulas.^[12]^ Discrepancies will be identified and resolved through discussion with other reviewers at each step of the study selection and data extraction process. Missing or insufficient data will be requested from the corresponding authors of the relevant studies.

### 2.4 Quality assessment

The methodological quality of the included studies will be evaluated using the Risk of Bias tools from the Cochrane Handbook version 6.1.0.^[13]^ This tool is used to assess bias in the domains of selection (random sequence generation and allocation concealment), performance (blinding of participants and personnel), detection (blinding of outcome assessors), attrition (incomplete outcome data), reporting (selective reporting), and other biases. Each domain will be ranked as low, uncertain, or high risk.

To assess the quality of evidence, the grading of recommendations assessment, development, and evaluation (GRADE) approach was used. Two independent researchers (GH and SJK) will evaluate each item, such as the risk of bias, inconsistency of results, indirectness of evidence, imprecision of the results, publication bias, large magnitude of an effect, and dose–response gradient.^[14]^

### 2.5 Data analysis and synthesis

#### 2.5.1 Analysis and synthesis strategy

Cochrane Collaboration Review Manager software (RevMan 5.4, Cochrane Collaboration, Oxford, UK) will be used to analyze the data. Dichotomous data will be presented as risk ratios with 95% confidence intervals, whereas continuous data will be expressed as means ± standard deviation. *I*^2^ statistics will be used to assess the statistical heterogeneity among pooled studies. If there is significant heterogeneity (I^2^ ≥ 50%), a random-effects model will be adopted. Otherwise, the fixed-effects model will be applied. A sensitivity analysis will be further performed when there is heterogeneity between pooled studies to rule out improper studies for reanalysis.

#### 2.5.2 Subgroup or subset analyses

Subgroup analyses will be performed according to the type of intervention (TSYB alone or TSYB combined with Western medication), the type of comparator group, or the type of IBS (diarrhea- or constipation-predominant).

### 2.6 Ethics and dissemination

There is no need to approve ethical issues because this study only involves a protocol for a systematic review. We will publish the results of the study in peer-reviewed journals and distribute them electronically or in print.

## 3 Discussion

Epidemiologically, IBS affects 10% to 15% of the population.^[15]^ Conventional Western medications have shown limited efficacy for the treatment of IBS, with repeated treatments required due to high recurrence rates, which leads to socioeconomic burden.^[16]^ Although TSYB has long been used to treat abdominal pain accompanied by diarrhea, its efficacy and safety for IBS have not been sufficiently investigated. A systematic review published in 2019 on the effect of TSYB in diarrhea-predominant IBS patients showed that TSYB was effective and safe compared to conventional medications and the placebo.^[17]^ However, these results require updating. Through this systematic review, we will investigate not only the sole effect of TSYB but also the synergistic effect of TSYB and conventional Western medicine or probiotics. This systematic review will also clarify the evidence on the efficacy and safety of TSYB as a complementary treatment for IBS, the results of which will be instructive for both IBS patients and health care providers.

## Acknowledgments

This study was supported by the project “Development of Korean medicine clinical practice guidelines” of the Guideline Center for Korean Medicine, National Institute for Korean Medicine Development (Project number: HF20C0051).

## Author contributions

Authorship: The protocol was conceptualized by GH and SJK. The study was drafted by GH and SJK. SJK and GH developed the search strategy. GH, SJK, KJK, and HJ will participate in study identification and exclusion, data extraction, and risk of bias assessments. Data syntheses and analyses will be performed by SJK and GH. JWP will arbitrate any disagreements and ensure that no errors are made in this review. All authors have read and approved the final manuscript.

**Conceptualization:** Gajin Han, Seok-Jae Ko, Jae-Woo Park.

**Funding acquisition:** Jae-Woo Park.

**Methodology:** Gajin Han, Seok-Jae Ko, Keumji Kim, Hyejin Jun.

**Supervision:** Seok-Jae Ko, Jae-Woo Park.

**Writing – original draft:** Gajin Han.

**Writing – review & editing:** Seok-Jae Ko, Jae-Woo Park.
